# Simultaneous cell growth and ethanol production from cellulose by an engineered yeast consortium displaying a functional mini-cellulosome

**DOI:** 10.1186/1475-2859-10-89

**Published:** 2011-11-01

**Authors:** Garima Goyal, Shen-Long Tsai, Bhawna Madan, Nancy A DaSilva, Wilfred Chen

**Affiliations:** 1 Department of Chemical Engineering, University of Delaware, Newark, DE 19716, USA; 2 Department of Chemical and Environmental Engineering, University of California, Riverside, CA 92521, USA; 3 Department of Chemical Engineering and Materials Science, University of California, Irvine, CA 92697, USA

**Keywords:** cellulose, cellulosome, ethanol, yeast, consolidated bioprocessing

## Abstract

**Background:**

The recalcitrant nature of cellulosic materials and the high cost of enzymes required for efficient hydrolysis are the major impeding steps to their practical usage for ethanol production. Ideally, a recombinant microorganism, possessing the capability to utilize cellulose for simultaneous growth and ethanol production, is of great interest. We have reported recently the use of a yeast consortium for the functional presentation of a mini-cellulosome structure onto the yeast surface by exploiting the specific interaction of different cohesin-dockerin pairs. In this study, we engineered a yeast consortium capable of displaying a functional mini-cellulosome for the simultaneous growth and ethanol production on phosphoric acid swollen cellulose (PASC).

**Results:**

A yeast consortium composed of four different populations was engineered to display a functional mini-cellulosome containing an endoglucanase, an exoglucanase and a β-glucosidase. The resulting consortium was demonstrated to utilize PASC for growth and ethanol production. The final ethanol production of 1.25 g/L corresponded to 87% of the theoretical value and was 3-fold higher than a similar yeast consortium secreting only the three cellulases. Quantitative PCR was used to enumerate the dynamics of each individual yeast population for the two consortia. Results indicated that the slight difference in cell growth cannot explain the 3-fold increase in PASC hydrolysis and ethanol production. Instead, the substantial increase in ethanol production is consistent with the reported synergistic effect on cellulose hydrolysis using the displayed mini-cellulosome.

**Conclusions:**

This report represents a significant step towards the goal of cellulosic ethanol production. This engineered yeast consortium displaying a functional mini-cellulosome demonstrated not only the ability to grow on the released sugars from PASC but also a 3-fold higher ethanol production than a similar yeast consortium secreting only the three cellulases. The use of more complex cellulosomal structures may further improve the overall efficiency for ethanol production.

## Background

It has been estimated that 1.3 billion mega-tons (dry weight) of terrestrial plants are produced annually on a world-wide basis [1]. Due to its renewable, abundant, and sustainable nature, lignocellulosic biomass is the only feedstock to potentially substitute for fossil fuels. Ethanol, which is generally expected to be the first major commercial product of this emerging cellulosic biofuel technology, has great potential to lessen our country's dependency on fossil fuel [2].

Unfortunately, the recalcitrant nature of cellulosic materials and the high cost of enzymes required for efficient hydrolysis are the major limiting steps to the more widespread exploitation of this natural resource [3]. Consolidated bioprocessing (CBP), which combines the production of enzymes, hydrolysis of cellulose, and fermentation of glucose and xylose to ethanol in one reactor, is gaining increasing recognition as a potential breakthrough for cellulosic ethanol production as up to a four-fold reduction in cost can be potentially achieved [2,4]. An ideal microorganism for CBP should possess the capability of efficient enzyme production and simultaneous cellulose saccharification and ethanol fermentation. *Saccharomyces cerevisiae *is an attractive candidate because of its high ethanol productivity and inherent ethanol tolerance [5]. In recent years, attempts have been made to engineer *S. cerevisiae *for cellulose hydrolysis under anaerobic conditions with only varying degrees of success [6-8].

Cellulosomes are naturally occurring elaborate enzyme complexes found in many anaerobic microorganisms that can efficiently hydrolyze cellulose based on the high level of enzyme-substrate synergy [9]. The synergistic effects are due to (1) the targeting effect of the cellulose binding module, (2) the proximity effect of the enzymes, and (3) the elimination of substrate inhibition from the quick uptake of glucose. We have recently reported the use of a yeast consortium for the functional presentation of a mini-cellulosome structure onto the yeast surface by exploiting the specific interaction of the different cohesin-dockerin pairs employed [10]. We demonstrated not only the feasibility and flexibility of the consortium system, but also the benefit of mini-cellulosomes to facilitate ethanol production. Unfortunately, direct ethanol production from phosphoric acid swollen cellulose (PASC) was achieved only using resting-cell cultures and the feasibility of simultaneous growth and ethanol production had not been demonstrated. In this paper, we demonstrate for the first time the use of this synthetic yeast consortium for direct growth and ethanol production from PASC, an important first step toward the ultimate goal of CBP. Quantitative polymerase chain reaction (qPCR) was used to investigate the dynamics of the individual populations during fermentation.

## Results and discussion

### Surface display of the mini-scaffoldin Scaf-ctf using the constitutive Agα1 anchor system

**Figure 1:**
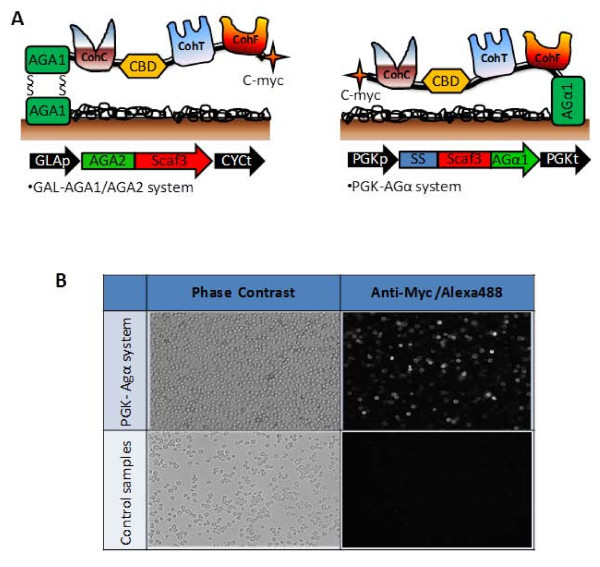
**Improved surface display of scaffoldin Scaf-ctf using the Agα1 anchor and the constitutive PGK promoter**. (A) Schematic representation of the two different surface display approaches. (B) Confirmation of surface displayed Scaf-ctf by immunofluorescence microscopy. Cells were probed with anti-Cmyc sera and fluorescently stained with a goat anti-mouse IgG conjugated with Alexa Fluor 488. Yeast cells harboring pCEL15 were used as the control.

To enable the direct growth and ethanol production on PASC by the synthetic yeast consortium, the Aga1-Aga2 anchor system used in the previous study [10] which required galactose for induced expression was replaced by a constitutively expressed Agα1 anchor system using a strong PGK promoter (Figure 1A). In addition, the entire expression cassette was transferred to a CEN/ARS-based plasmid (YCplac33-AGα-scaf3) containing the centromeric sequence to ensure a constant copy number and improved protein expression. This plasmid was subsequently transformed into *S. cerevisiae *strain BY4742, which was then denoted as strain SC.

To demonstrate the display of Scaf-ctf, immunofluorescence assays were carried out using the anti C-myc antibody (Figure 1B). A detectable fluorescence signal was observed for over 85% of cells, which is higher than the 60% observed for cells using the Aga1-Aga2 anchor system. This improved percentage of surface display can be attributed to the improved plasmid stability using the CEN/ARS-based plasmid and the reported superior display efficiency of the Agα1 anchor system [11].

### Growth and ethanol production from PASC

**Figure 2:**
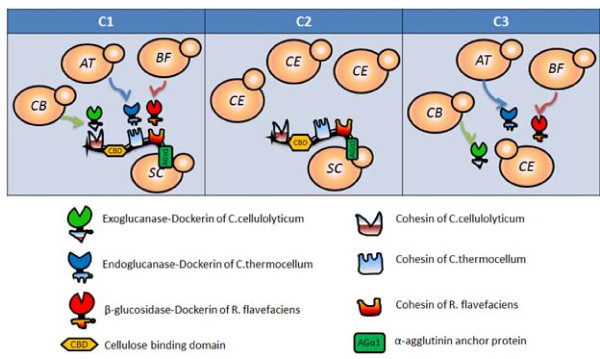
**A schematic of the different consortia used in this study**.

The ability of the consortium to grow and produce ethanol directly from PASC was investigated. In addition to the newly constructed strain displaying Scaf-ctf (SC) under a constitutive promoter, three other strains secreting either an endoglucanase (AT), an exoglucanase (CB) or a β-glucosidase (BF) tagged with a different dockerin domain and flanked by a His6 tag used in the consortium were as described before (Table 1) [10]. Different yeast strains were initially grown separately in SDC medium overnight and then mixed in the optimized ratio (7:2:4:2) to a total initial cell density of 8 × 10^6 ^cell/ml to form the functional consortium (C1) [12]. A strain carrying the plasmid pCEL15 (CE) with no heterogenous protein expression was used as a control population (Table 1). To compare the performance, two other consortia composed of either only the Scaf-ctf-displaying cells (SC) and CE (C2) or cellulase-secreting cells (AT/CB/BF) and CE (C3) at the same ratio as C1 were used. All consortia developed are depicted in Figure 2.

**Table 1:** Strains and plasmids used in this study.

Strain	Plasmid	Phenotype	Source
**CE**	pCEL15	Secretes a small peptide (negative control)	Tsai *et al*, 2010
**AT**	pAt	Secretes the endoglucanase At (CelA from *C. thermocellum *with its native dockerin)	Tsai *et al*, 2010
**CB**	pCBH2c	Secretes the cellobiohydrolase CBHc (CBHII from *T. reesei fused *with a dockerin from *C. cellulolyticum*)	Tsai *et al*, 2010
**BF**	pBGLf	Secretes the β-glucosidase Bglf (Bg1I from *T.aurantiacus *fused with a dockerin from *R. flavefaciens*)	Tsai *et al*, 2010
**SC**	pAgα-scaf3	Display of Scaf-ctf by an Agα1 anchor in a centromeric plasmid	This study

*S. cerevisiae *strain BY4742 was used in all cases.

**Figure 3:**
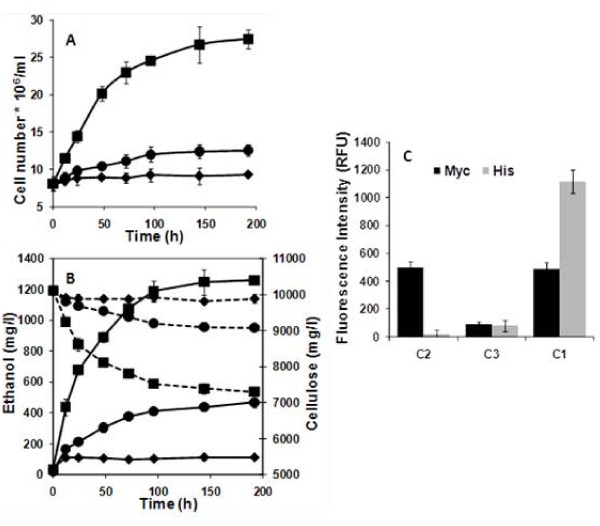
**Cell growth and ethanol production by the cell consortia**. (A) Cell growth and (B) PASC hydrolysis (dotted line) and ethanol production (solid line) by the different yeast consortia, i.e., consortium C2 without secreting enzymes (♦), consortium C3 only secreting enzymes (●) and consortium C1 forming the cellulosome structure (■). (C) Surface display of the mini-cellulosome was probed with either anti-C-myc sera for the displayed scaffoldin or anti-C-His6 sera for the three cellulases docked on the scaffoldin and fluorescently stained with a goat anti-mouse IgG conjugated with Alexa Fluor 488. Whole cell fluorescence was determined using a fluorescent microplate reader. Data shown are the mean values (± standard deviation) obtained from 3 independent experiments.

An initial glucose concentration of 0.2 g/L was added to allow the synthesis and assembly of the cellulosome structure. For the consortium C2 containing only SC, no appreciable level of cell growth and PASC degradation was observed; only the added glucose was converted to ethanol (Figure 3A and 3B). In comparison, a significant level of cell growth was observed for the consortium C1 containing the functionally displayed cellulosome, and only minimum growth was detected for the consortium C3 secreting only cellulases (Figure 3A). The enhancement in cell growth was also reflected in both PASC degradation and ethanol production; the final ethanol level of 1.25 g/L is 3-fold higher than the consortium secreting only cellulases (Figure 3B). The final ethanol yield of 0.43 g ethanol/g PASC is equivalent to 87% of the theoretical value. Even though the ethanol productivity is much lower than required in practice [13], our results successfully demonstrated the concept of using a microbial consortium for the simultaneous growth and ethanol production from cellulose. However, further improvements of the consortium system are required to significantly improve the overall productivity.

### Verification of mini-cellulosome assembly

Whole cell fluorescence measurements were undertaken to verify and quantify the assembly of secreted cellulases onto the cell surface. Cells were harvested after fermentation and washed three times with buffer before probing with both anti-Cmyc (Scaf-ctf) and anti-His antibodies (his tagged cellulases). As shown in Figure 3C, the correct assembly of the mini-cellulosome was observed only for consortium C1, which showed an appreciable level of whole-cell fluorescence toward both antibodies, indicating the simultaneous display of Scaf-ctf, and docking of cellulases. In contrast, fluorescence was detected only with the Cmyc antibody for consortium C2 displaying only Scaf-ctf and only background levels of fluorescence were observed with consortium C3 (Figure 3C). These results are significant as they demonstrate, for the first time, that a synthetic consortium can be successfully engineered for the functional display of cellulosomes for cellulosic ethanol production in a CBP-like setting.

### Dynamics of the yeast consortium by qPCR

**Figure 4:**
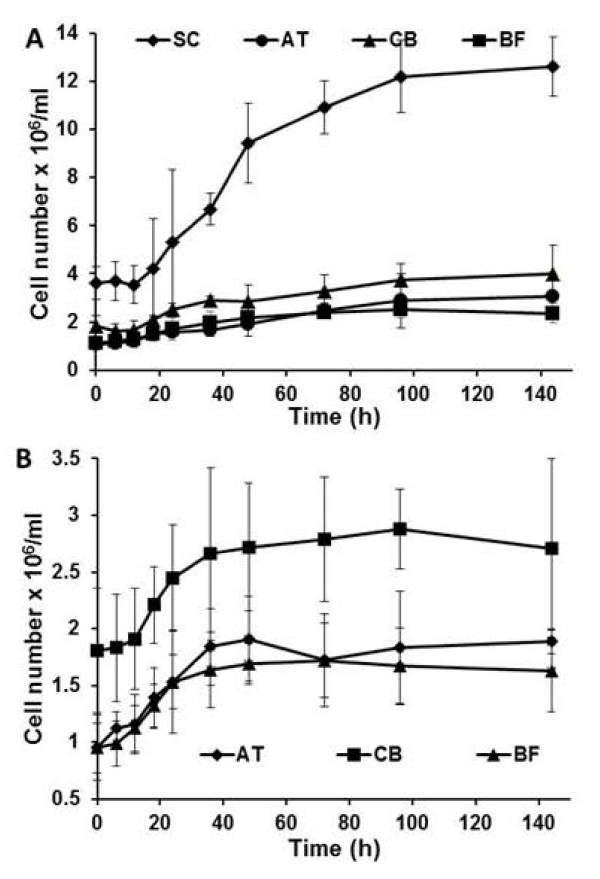
**Growth dynamics of individual populations in (A) consortium C1 that could form cellulsosome structure and (B) consortium C3 that can only secret enzymes**. Changes in cell number of individual yeast populations were probed by qPCR. Data shown are the mean values (± standard deviation) obtained from 3 independent experiments.

To gain a better understanding of the synthetic yeast consortium, qPCR was used to probe the dynamics of all four yeast populations during fermentation. Primers (Table 2) were designed to target a ~250 bp region of a unique gene of each population coding for either the endoglucanase (AT), the exoglucanase (CB), the β-glucosidase (BF), or the displayed Scaf-ctf (SC). Total DNA was individually extracted from the four different populations and a linear standard curve spanning three-log concentrations from 10^4 ^to 10^6 ^cell/ml was generated under optimized conditions (Data not shown). Using the qPCR method, cell growth was clearly demonstrated for all four populations during fermentation (Figure 4). However, the final cell density increased by over 3-fold for the strain displaying the functional mini-cellulosome (SC), while roughly a 2-fold increase was observed for the other three populations. This difference in the growth rates is consistent with other reports indicating that the use of a ternary cellulose-enzyme-microbe complex (SC) yields much higher rates of cellulose utilization than using only a cellulose-enzyme complex (AT, CB, and BF) [14]. It is interesting to note that strains secreting enzymes (AT, CB, BF) were growing at slightly different rates probably a result of dissimilar levels of metabolic burden due to expression of different cellulases. Even with the differences in growth, the final population ratio of 7:1.8:3.4:1.9 did not change significantly from the initial inoculation ratio. It should be noted that the growth curve obtained using the qPCR method was in 90% agreement with the results obtained from direct cell counting, indicating the validity of the qPCR method to rapidly track the temporal dynamics of the individual population during fermentation.

**Table 2:** Primers used in this study

Primers	Sequence (5'-3')	Relevance
**PgkFp**	CCGCCATGGTGTTTGCAAAAAGAACAAAACTG	Subcloning of Agα-Scaf
**PgkRp**	CCGCCATGGCCCTATGCGGTGTGAAATACC	Subcloning of Agα-Scaf
**Fxba1-Sctf**	GCGCTCTAGAGGCGATTCTCTTAAAGTTACAGT	Subcloning of Agα-Scaf
**ScafFP**	GCGCCAAAAGCTCTTTTATCTCAACC	qPCR
**ScafRP**	CCACATCACTAATCACTTCTGATGTGGTG	qPCR
**AtFP**	GCAGAATGGGAAGACTGGAAGAGC	qPCR
**AtRP**	CCGCCGTCATGACTTGTAACATTGTTG	qPCR
**CBHIIFP**	CGCAAAGGTTCCCTCTTTTATGTGGC	qPCR
**CBHIIRP**	TCCGGATATCGGAATATTCCACGACAA	qPCR
**BglfFP**	ATCATGGCGGCCTTTTACAAGGTTG	qPCR
**BglfRP**	CCTCTCCAAAAACTCCGGTGAACTTTTC	qPCR

Similarly, the dynamics of consortium C3 secreting only cellulases was probed using qPCR (Figure 4). Again, all three enzyme-secreting strains (AT, CB, and BF) were shown to grow during the fermentation. Although cell growth for all three strains was slower than in consortium C1, the total final cell density of the three strains was ~85% of that of consortium C1. This slight reduction in the cell density cannot explain the observed 3-fold difference in PASC hydrolysis and ethanol production. Instead, the substantial increase in ethanol production is consistent with our previously reported synergistic effect on cellulose hydrolysis using the displayed mini-cellulosome structure when compared with free enzymes [10].

## Conclusions

In this era of high energy demand, there is an urgent need to develop new cost-effective methods that can convert complex cellulosic biomass into simple sugars and eventually ethanol. Our group has recently mimicked the natural anaerobic cellulose degradation mechanism by displaying a mini-cellulosome on the yeast surface and observed a similar synergistic effect on cellulose hydrolysis and ethanol production compared to free enzymes [10,12]. To accomplish the goal of simultaneous cell growth and ethanol production on cellulose, we engineered a yeast consortium capable of the surface assembly of a functional mini-cellulsome via intercellular complementation. The resulting consortium can grow on cellulose and produce ethanol more efficiently than a similar consortium secreting only cellulases because of the synergistic action on cellulose hydrolysis by the mini-cellulosome structure. Although the level of ethanol production is relative modest, this is a promising first step toward the goal of CBP using an engineered yeast consortium. Further improvements in the overall productivity necessitate the use of more complex cellulosome structures as in natural anaerobic microorganisms. The flexibility of the consortium design offers the possibility of displaying more complex cellulosomes by manipulating the individual population involved in the consortium.

## Methods

### Strains, plasmids, and media

*Escherichia coli *strain JM109 [*rec*A1 *end *A1 *sup*E44 *hsd*R17 *gyr*A96 *thi*, *rel*A1, λ^-1 ^Δ(*lac*-*pro*AB) F *tra*D36 *pro*AB *lac*IqZ DM15] was used as a host for genetic manipulations. Cells were grown in LB medium (5 g/l yeast extract, 10 g/l NaCl, 10 g/l tryptone) supplemented with ampicillin (100 mg/l) when required. *S. cerevisiae *strain BY4742 (*MAT***α ***his3*Δ*1 leu2*Δ*0 lys2*Δ*0 ura3*Δ*0*) was used for displaying the scaffoldin and secretion of cellulases. The phenotypes and sources of the yeast strains and plasmids that were used in this study are listed in Table I. Yeast strains were routinely cultured in SDC medium (20 g/l dextrose, 6.7 g/l yeast nitrogen base, and 5 g/l casamino acids) at 30°C on a rotary shaker at 250 rpm.

### Construction of YCplac33-AGα-Scaf3 for constitutive surface-display of Mini-scaffoldin Scaf-ctf

A centromeric plasmid, YAGα-Scaf3, used for surface display of the trifunctional mini-scaffoldin Scaf-ctf, was constructed as described below. All primers used in cloning are given in Table 2. The Scaf-ctf fragment, consisting of three different cohesins from *Clostridium cellulolyticum*, *Clostridium thermocellum and Ruminococcus flavefaciens *and a cellulose binding module (CBM), was amplified from the plasmid pSctf [10] by PCR using primers FXba1-Sctf and Sctf-Sal1R. The resulting fragment (2046 bps) was digested with *Xba*1 and *Sal*1 and cloned into the *Xba*1 and *Sal*1 sites of a multiple copy surface-display vector pSSAGα, which consisted of the yeast 3-phosphoglycerate kinase (PGK1) promoter, the secretion signal of *Rhizopus oryzae *amylase, a C-myc tag, the C-terminus α-agglutinin gene AGα1 and the PGK1 terminator. The resulting plasmid was named pAGα-Sctf. The entire expression cassette encoding the PGK promoter to the PGK terminator was then amplified from plasmid pAGα-Sctf by PCR using primers PgkFp and PgkRp. The PCR product obtained was then subcloned into the *Sma*1 site of the CEN/ARS-based vector YCplac33 via blunt end ligation after kinase treatment. Transformants were confirmed by restriction digestion and named YCplac33-AGα-scaf3. The YCplac33-AGα-scaf3 plasmid was transformed in *S. cerevisiae *BY4742 using the standard lithium acetate procedure [15].

### Anaerobic fermentation

PASC was prepared as described by Walseth from Avicel PH101 (Sigma) [16]. For anaerobic fermentation, different consortia were grown in rubber stoppered glass serum bottles containing SC-PASC medium (6.7 g/l yeast nitrogen base w/o amino acids, 20 g/l casamino acids, and 10 g/l PASC supplemented with 10 mM CaCl_2_, 0.01 g/l ergosterol and 0.42 g/l tween 80). Precultures of each yeast population were grown separately in SDC media (20 g/l glucose, 6.7 g/l yeast nitrogen base, 5 g/l casamino acids), harvested, and washed with sterile water to prevent media carry over. For co-culturing of the synthetic consortia, each strain was mixed initially in the optimized ratio to a total optical density of 0.8. Samples were collected periodically through a capped syringe needle pierced through the bottle stopper [12]. Yeast cells in fermentation media were counted in triplicate on SDC plates by the plate count method.

### Reducing sugar and ethanol assays

Reducing sugars were measured by the DNS method. Samples were collected periodically and mixed immediately with equal amount of DNS reagents (10 g/l dinitrosalicylic acid, 10 g/l sodium hydroxide, 2 g/l phenol, 0.5 g/l sodium sulfite) and incubated for 5 to15 min at 95°C. 1 ml of 40% Rochelle salts was added to fix the color before measuring the absorbance at 575 nm using a spectrophotometer. The glucose concentration was determined by using a Sigma HK assay kit. For measuring the amount of unhydrolyzed cellulose, the phenol-sulfuric acid method described by Dubois et. al. was used [17]. Ethanol concentration was measured using a gas chromatograph (model 6890, Hewlett Packard, USA) with a HP-FFTP column and a flame ionization detector (FID) detector.

### Immunofluorescence assay

Immuno-fluorescence microscopy was done as described previously [10]. In short, cells were washed with PBS (phosphate buffered saline) and resuspended in PBS containing 1 mg/ml BSA (bovine serum albumin). Either anti-His6 or anti-Cmyc antibody were added and incubated at room temperature for 1 h on a rotary shaker. After washing, AlexaFluor™488 - conjugated anti-mouse secondary antibody was added. Cells were then washed three times with PBS buffer and resuspended in PBS buffer mixed with 1 mg/ml BSA. Whole-cell fluorescence images were obtained using a fluorescence microscope (Olympus BX51) with an excitation wavelength at 485 nm and an emission wavelength at 535.

### Real time quantitative PCR

Total DNA from each strain was extracted using the High Pure PCR Template Preparation Kit (Roche Applied Science, Germany), and the concentration was determined using a Nano-drop spectrophotometer at 260/280 nm. All primers used for PCR reactions are listed in Table II. Quantitative PCR assays were done in 25 μl final volumes containing 2 μl DNA template, 0.2 μM each respective primer, and 12.5 μl of SYBR Green Master Mix (Fisher Scientific). All amplifications were carried out in optical grade 96 well plates from Bio-rad with an initial step at 95°C for 3 min followed by 40 cycles of 95°C for 15 s, 57°C for 1 min, 72°C for 30 s. All samples were triplicated in culture and analysis. To quantify the individual yeast population, a standard curve was generated for each individual cell population by ten-fold dilutions from 10^6 ^CFU/ml to 10^4 ^CFU/ml.

## Abbreviations

CBM: cellulose binding module; CBP: consolidated bioprocessing; FID: flame ionization detector; PASC: phosphoric acid swollen cellulose; PBS: phosphate buffered saline; PCR: polymerase chain reaction; qPCR: quantitative polymerase chain reaction.

## Competing interests

The authors declare that they have no competing interests.

## Authors' contributions

WC developed the idea for the study. WC, ND and ST design the research. GG and ST did the literature review and prepared the manuscript. ST and BM constructed the vectors. GG did the majority of the lab work, cultivations and enzyme essays. Figures were prepared by GG and ST. WC and ND supervised the study, and participated in the design and coordination and helped to revise the manuscript. All authors read and approved the final manuscript.
